# Old poisons, new signaling molecules: the case of hydrogen cyanide

**DOI:** 10.1093/jxb/erad317

**Published:** 2023-08-16

**Authors:** Pablo Díaz-Rueda, Laura Morales de los Ríos, Luis C Romero, Irene García

**Affiliations:** Instituto de Bioquímica Vegetal y Fotosíntesis (IBVF), CSIC-Universidad de Sevilla, 41092-Sevilla, Spain; Instituto de Bioquímica Vegetal y Fotosíntesis (IBVF), CSIC-Universidad de Sevilla, 41092-Sevilla, Spain; Instituto de Bioquímica Vegetal y Fotosíntesis (IBVF), CSIC-Universidad de Sevilla, 41092-Sevilla, Spain; Universidad de Sevilla, Spain

**Keywords:** Hydrogen cyanide, immune response, metalloproteins, plant defense, *S*-cyanylation, signaling

## Abstract

The high phenotypic plasticity developed by plants includes rapid responses and adaptations to aggressive or changing environments. To achieve this, they evolved extremely efficient mechanisms of signaling mediated by a wide range of molecules, including small signal molecules. Among them, hydrogen cyanide (HCN) has been largely ignored due to its toxic characteristics. However, not only is it present in living organisms, but it has been shown that it serves several functions in all kingdoms of life. Research using model plants has changed the traditional point of view, and it has been demonstrated that HCN plays a positive role in the plant response to pathogens independently of its toxicity. Indeed, HCN induces a response aimed at protecting the plant from pathogen attack, and the HCN is provided either exogenously (*in vitro* or by some cyanogenic bacteria species present in the rhizosphere) or endogenously (in reactions involving ethylene, camalexin, or other cyanide-containing compounds). The contribution of different mechanisms to HCN function, including a new post-translational modification of cysteines in proteins, namely *S*-cyanylation, is discussed here. This work opens up an expanding ‘HCN field’ of research related to plants and other organisms.

## Teaser

Hydrogen cyanide is a signaling molecule that regulates plant development and response to pathogens through different mechanisms, including protein modifications by *S*-cyanylation.

## Introduction

As sessile organisms, plants are unable to move to avoid adverse conditions. Therefore, they evolved extremely efficient mechanisms for detecting and responding to the wide diversity of biotic and/or abiotic stress conditions to restore cellular homeostasis.

The detection of changes in external conditions acquires special relevance and, concomitantly, the signaling mechanisms triggering responses aimed at restoring homeostasis or adapting their physiology to the new conditions are extremely efficient. The molecular mechanisms that underlie cell signaling in plants constitute a central topic of research, especially in our current climatic change scenario. Among them, the role of small signal molecules deserves attention because they are able to trigger rapid responses mainly due to their high chemical reactivity.

Hydrogen cyanide (HCN) is a well-known poison used since ancient times for suicides and murders, including mass killings. Additionally, industrial wastes and leakages of HCN are the origin of environmental pollution and catastrophes. It has been extensively referred to in detective books, where it often appears as a substance with the characteristic odor of bitter almonds. It is a low molecular weight molecule that is highly reactive, soluble in water, and has a low melting point. Due to its reactivity and its abundance in the Earth’s earliest atmosphere, the participation of HCN in the origin of ribonucleotides, lipids, and amino acids is more than possible (Patel *et al.*, 2015). Its toxic capacity is mainly due to its ability to form very stable complexes with transition metals of the prosthetic groups of metalloproteins that are essential for their function (Nagahara *et al.*, 1999). The main target of cyanide in living organisms is the mitochondria, where it blocks electron transfer by cytochrome *c* oxidase and thus interrupts mitochondrial oxygenic respiration (Donato *et al.*, 2007), although it also affects photosynthetic enzymes in chloroplasts (Berg and Krogmann, 1975) and the activity of enzymes such as catalase and oxidases (Cheeke, 1995; McMahon *et al.*, 1995)

Therefore, HCN has been considered only as a toxic compound whose presence is rapidly eliminated by detoxifying activities. However, its physicochemical characteristics are similar of those of other low molecular weight signaling molecules, such as nitric oxide (NO) and hydrogen sulfide (H_2_S), which are toxic at high concentrations and possess, at non-toxic concentrations, a widely demonstrated and accepted signaling role in plants (Delledonne *et al.*, 2001; Astier and Lindermayr, 2012; Paul and Snyder, 2015; Aroca *et al.*, 2018).

### HCN in life kingdoms

**Fig. 1.**
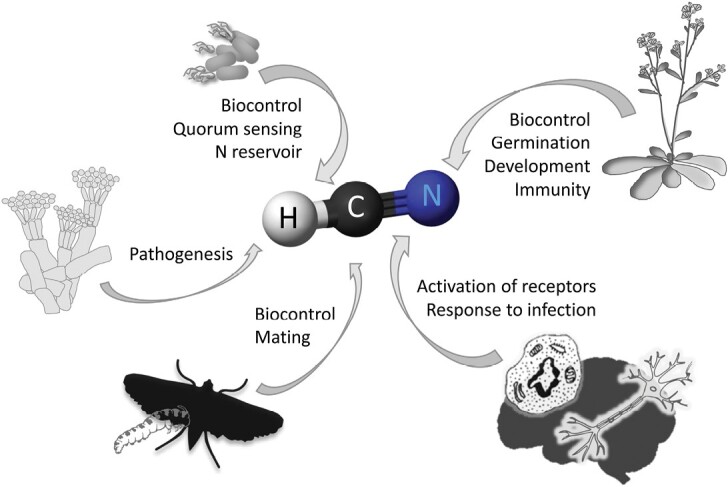
HCN functions in organisms of the different kingdoms of life. Despite its toxicity, HCN is present in living organisms, where functions have been described. The different functions in the different life kingdoms are indicated adjacent to the respective arrows. For explanations and references, see the text.

HCN has been described to be synthetized within cells of organisms from all kingdoms except *Archaea*. In bacteria and fungi, the amino acid glycine is oxidized and decarboxylated by the membrane-bound flavoenzyme cyanide synthase, giving HCN and CO_2_ as products (Knowles, 1976, 1988; Blumer and Haas, 2000). In these organisms, HCN and HCN-containing molecules such as cyanogenic glucosides can serve as a nitrogen source for amino acids and other N-containing molecules, but other roles for HCN have been identified or suggested (Fig. 1). Some HCN-emitting soil bacterial strains, especially fluorescent pseudomonads, have important effects against plant diseases and, historically, this effect has been attributed to a direct poisonous effect. However, it has also been shown that these bacteria stimulate plant growth depending on their capacity to emit HCN (Kuzmanovic *et al.*, 2018; Sehrawat *et al.*, 2022; see below). In the context of animal–bacteria interactions, it is interesting to mention the case of *Pseudomonas aeruginosa*, an opportunistic human pathogen affecting the lungs and causing pneumonia. A wide range of *P. aeruginosa* strains produce HCN, which has been shown to be involved in the process of lung tissue colonization by quorum-sensing mechanisms by inducing the production of biofilm components and by competition with other bacterial species, such as *Staphylococcus aureus*, not only in proximity but also at a distance (Létoffé *et al.*, 2022).

Cyanogenesis in arthropods has also been described. In many cases, arthropods accumulate cyanogenic compounds from the plants they feed on, and they prefer cyanogenic rather than non-cyanogenic plants to feed on (Zagrobelny *et al.*, 2007, 2008), but some arthropods are able to synthesize cyanogenic glucosides *de novo* as well (Siegien and Bogatek, 2006; Zagrobelny *et al.*, 2008). In addition to defense, a pheromone-like function has been described for HCN-producing compounds in mating behaviors, such as male courtship and female calling and assessment of male fitness (Fig. 1) (Zagrobelny *et al.*, 2018).

Mammalian cells are able to produce HCN from glycine in a reaction catalyzed by peroxidases (Stelmaszynska, 1986; Borowitz *et al.*, 1997; Gunasekar *et al.*, 2000). Compared with plants and bacteria, HCN signaling and regulatory function in mammals are paticularly unknown and underestimated because it is almost exclusively viewed as a poison and environmental toxin. Nevertheless, mammals, including humans, have detectable (nM to low µM) plasma HCN levels (Vinnakota *et al.*, 2012), and active white blood cells produce HCN during phagocytosis (Stelmaszynska, 1986). In the brain, synaptic receptors are activated by HCN, which is generated by opiate agonists, indicating that it may act as a neuromodulator (Fig. 1) (Borowitz *et al.*, 1997; Gunasekar *et al.*, 2004; Siegien and Bogatek, 2006). It has been observed recently that very low concentrations of HCN (from nM to 1 µM), rather than having toxicological effects, induce cellular proliferation and bioenergetics via cytochrome *c* oxidase stimulation and increase cell respiration and ATP production in mammalian cell cultures (Pacher, 2021; Randi *et al.*, 2021).

### HCN in plants

HCN is naturally present in relatively high concentrations in cyanogenic plant species such as almonds or cassava (Poulton, 1990; Moller, 2010), forming cyanogenic compounds that, in the case of the latter, can generate a health problem if not properly eliminated, especially in developing countries in times of famine (White *et al.*, 1998; Kashala-Abotnes *et al.*, 2019). In non-cyanogenic plants, HCN is produced principally during the biosynthesis of ethylene (ET) and the antipathogenic molecule camalexin (Peiser *et al.*, 1984; Yip and Yang, 1988; Wang *et al.*, 2002; Glawischnig, 2007; Bottcher *et al.*, 2009). Therefore, HCN is naturally produced in plants and, apart from its toxic action, it has been shown to influence several physiological processes.

Substantial evidence has related the presence of HCN to the acceleration of germination and, indeed, the HCN content in seeds increases just before germination in many cyanogenic and non-cyanogenic plant species (Esashi *et al.*, 1991). It is well known that cyanohydrins present in the smoke of a wildfire induce a germination burst due to the HCN released, which regenerates the burned landscape (Nelson *et al.*, 2012; Flematti *et al.*, 2013). Treatments with HCN at low concentrations (mM) stimulate seed germination by breaking dormancy in diverse plants, including fruit trees, cereals, sunflower, and Arabidopsis, concomitantly with transient reactive oxygen species (ROS) production and protein carbonylation, and, in some cases, the ET signaling pathway has been shown to be necessary for dormancy break (Gotor *et al.*, 2019, and references therein). An RNA-seq analysis of tomato seeds treated with a low concentration of KCN has shown that HCN acts pleiotropically to break seed dormancy (Yu *et al.*, 2022). Specifically, repression of proteins necessary for seed protein storage that may cause mobilization of stored proteins, as well as induction of glycolytic, tricarboxylic acid (TCA) cycle, and oxidative phosphorylation enzymes, was observed. Therefore, carbon and energy metabolism acceleration is occurring. Finally, hormones such as abscisic acid and gibberellin were also affected in KCN-treated tomato seeds.

Hormones, including ET, are central components of the plant response to pathogens (Zhang *et al.*, 2018). As ET and HCN biosynthesis are intimately linked, a relationship between HCN and plant immunity is not unlikely and will be discussed below.

### HCN-mediated signaling in plants

Plants possess two HCN-detoxifying enzymatic activities, namely β-cyanoalanine synthases (CASs; EC 4.4.1.9) and sulfurtransferases (STRs; EC 2.8.1.1), which use either cysteine or thiosulfate/mercaptopyruvate to incorporate HCN and convert it into less toxic molecules. The main HCN-detoxifying enzyme in *Arabidopsis thaliana* is CAS, with mitochondria-localized CAS-C1 activity accounting for ~70% of the total CAS activity in this plant (Hatzfeld *et al.*, 2000; Machingura *et al.*, 2016; Arenas-Alfonseca *et al.*, 2018b). Although *cas-c1* T-DNA insertion mutants accumulate between 20% and 40% more HCN in their tissues than wild-type plants, their only phenotypic difference is that they present dwarf root hairs that are unable to elongate (Garcia *et al.*, 2010). Moreover, it has been shown that this defect is not dependent on ROS production or the inhibition of the NADPH oxidase RHD2 that produces the superoxide anion at the tip of the elongating root hair and drives its polar growth (Arenas-Alfonseca *et al.*, 2018a, b). On the other hand, the mutation in *CAS-C1* activates the immune response of plants when infected by bacterial and viral biotrophic pathogens, indicating that endogenously produced HCN might also contribute to regulating the plant immune system (Garcia *et al.*, 2013; see below). Overexpression of CAS genes in Arabidopsis has also been shown to be important in salt stress resistance, and alternative oxidase (AOX) seems to be essential in this process (Xu *et al.*, 2023).

HCN can thus act as a signal molecule whose amount is finely regulated in plant tissues and whose action mechanism is important to uncover. It is a small molecule able to penetrate membranes easily due to its lipid solubility. It is enzymatically generated and toxic in excess; therefore, detoxifying activities are necessary to maintain HCN at non-toxic levels. These features are shared by known gasotransmitter signaling molecules, such as ROS, and reactive sulfur and nitrogen species. Their mode of action includes post-translational modifications (PTMs) in proteins at the -SH groups of cysteines, namely oxidation, persulfidation, and nitrosylation (Aroca *et al.*, 2018, and references therein). PTMs influence the physicochemical properties of the proteins (folding, conformation, subcellular distribution, stability, and activity) and therefore their biological activity. As will be discussed below, HCN *per se* is capable of reacting with cysteine residues in the form of a disulfide bridge to form an organic thiocyanate (Gawron, 1966). We have shown very recently for the first time in any organism that *S*-cyanylation exists naturally in plants (Garcia *et al.*, 2019). The importance of this new PTM is unexplored and represents an exciting challenge. Box 1 summarizes the synthesis, detoxification, and signaling role of HCN in plants, including the *S*-cyanylation as a novel HCN-driven mechanism of action.

Box 1. **Synthesis, detoxification, and signaling role of HCN in plants.**

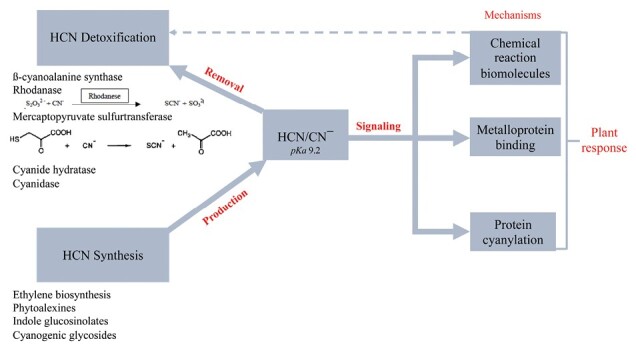

The intracellular levels of HCN are determined by its production as a co-product in the biosynthesis of ethylene and phytoalexins or in the hydrolysis and degradation of glucosinolates and cyanogenic glycosides, in equilibrium with the mechanism of enzymatic detoxification or its reaction with other biomolecules. The transient or permanent accumulation of HCN induces its reaction with small biomolecules or with proteins and enzymes, altering its functionality and acting as a signaling molecule in the regulation of pathophysiological processes in the plant.

Hence, HCN plays a role in different biological processes in plants, which deserves special attention because it has driven a change in the understanding of HCN from a poison to a signaling molecule. These findings are of special relevance because they represent a new mechanism for triggering fast and efficient responses aimed at restoring homeostasis and/or adapting plant physiology to a changing environment, especially important in the current climatic change scenario and the emergence of new plant diseases.

## HCN in plant defense

Cyanogenic compounds constitute an excellent defensive barrier against animal, microbial, and fungal attack, and exist in >3000 plant species. The core of plant cyanogenesis is based on the enzymatic hydrolysis of cyanohydrins (α-hydroxynitriles) to generate carbonyl compounds (aldehyde or ketone) with free toxic HCN as a consequence of tissue disruption triggered by herbivores or by physical injury (Morant *et al.*, 2008).

The cyanogenesis process in cyanogenic plants has been established by numerous studies, and a canonical pathway has been proposed: the synthesis of cyanogenic glycosides starts with the conversion of aromatic (Phe, Tyr) or aliphatic (Val, Ile, Leu, Trp) amino acids into the respective oximes catalyzed by cytochromes P450 of the CYP79 family (CYP79D1/D2/A1). Consecutively, oximes are converted into α-hydroxynitriles by the action of cytochromes P450 of the CYP71, CYP736, CYP706, and CYP83 families, to be then glycosylated by UDP-glycosyltransferases (UGT85B/K). CNglcs are formed as inactive precursors and need to be enzymatically activated. After tissue disruption mediated by feeding herbivores, CNglcs are brought into contact with β-glucosidases that break the β-glucosidic bond, thereby releasing α-hydroxynitriles (cyanohydrins) with defensive properties (Zagrobelny *et al.*, 2004). Finally, cyanohydrins are hydrolyzed either spontaneously or by hydroxynitrile lyases (HNLs), dissociating into HCN and the corresponding aldehyde or ketone (Gleadow and Møller, 2014).

HCN and cyanohydrins are too reactive to be deployed directly as pre-formed defenses. The storage of inactive precursor CNglcs permits the generation of appropriate chemically active compounds against herbivores in the right tissue and at the right time: activating enzymes that are compartmentalized separately to their substrates so that they do not mix until the plant has been chewed or damaged (Miller and Conn, 1980; Poulton, 1990; Conn, 2008; Moller, 2010; Gleadow and Møller, 2014). Although CNglcs are the most common cyanogenic compounds, derivatives from the 4-OH-ICN pathway exclusively found in the *Brassicaceae* family also produce cyanohydrins, which can then be hydrolyzed by the action of the HNL enzyme, releasing HCN (Rajniak *et al.*, 2015; Pastorczyk *et al.*, 2020). In the initial step of this pathway, two redundant P450 monooxygenases, CYP79B2 and CYP79B3, convert tryptophan into indole-3-acetaldoxime (IAOx), which then acts as the substrate taken by CYP71A12 to produce indole cyanohydrin. At this point, a flavin-dependent oxidoreductase, termed FOX1, catalyzes the conversion of IAOx to indole-carbonylnitrile (ICN), a substrate of the CYP82C2 enzyme that can be transformed into 4-OH-ICN. Additionally, 4-OH-ICN is the base for downstream cyanohydrin metabolite production (Dixit *et al.*, 2022).

The role of cyanogenic plants in the control of phytophagous arthropod pests has been widely studied (Boter and Diaz, 2023, and references therein). In general, insect damage negatively correlates with cyanogenic and phenolic compounds, and some studies have even demonstrated that insects such as Mexican bean beetles (*Epilachna varivestis*) choose plants with genotypes deficient in cyanide release as a source of nutrients or as a place to lay their eggs (Balhorn *et al.*, 2006). Recent studies provide evidence that the benefits of HCN defense against herbivory on white clover (*Trifolium repens*) are temperature dependent (Fadoul *et al.*, 2023). In contrast, there is just one publication validating the alternative cyanogenic pathway to expand plant defenses against pests in a non-cyanogenic plant such as *A. thaliana*. Arnaiz *et al.* (2022) demonstrated the up-regulation of different enzymes, such as β-glucosidases, α-HNLs, and FOX1, for the release of HCN in response to phytopredators such as the spider mite (*Tetranychus urticae*). Moreover, a reduction in leaf damage determined in the AtHNL-overexpressing lines reflected the mites’ reduced ability to feed on leaves, which consequently limited mite fecundity. Interestingly, it was recently identified how the interaction of plant–predator co-evolution has led to an arms race where insects induce β-cyanoalanine synthase for HCN detoxification as protection against the defenses of Arabidopsis plants (Dixit *et al.*, 2022; Nguyen, 2022).

HCN has also been proposed as a signaling molecule that could act indirectly in the plant response to pathogenic bacteria, viruses, or fungi. *PR-1* and several salicylic acid (SA)-regulated genes are transcriptionally induced in the basal state in *cas-c1* mutant plants (Garcia *et al.*, 2013, 2014). It has been extensively demonstrated that *cas-c1* mutant plants are more resistant than wild-type plants to biotrophic and hemibiotrophic pathogens such as the bacterium *Pseudomonas syringae* and beet curly top virus (BCTV) (Garcia *et al.*, 2013, 2014). Furthermore, double mutants *atrbohD3 cas-c1* show a notably lower susceptibility to *P. syringae* pv. tomato DC3000 infection than the simple mutant *atrbohD3*, suggesting that the accumulation of HCN due to the *cas-c1* mutation is capable of activating plant defenses in a similar way to the effect of ROS produced by NADPH oxidase (Arenas-Alfonseca *et al.*, 2021). Interestingly, the *cas-c1* mutant does not produce more O_2_^–^ than wild-type plants; therefore, the bypass of NADPH oxidase does not depend on ROS production in the presence of HCN (Arenas-Alfonseca *et al.*, 2021). Proteomic analyses of wheat and barley varieties with different sensitivities to the fungus *Fusarium graminearum* reveal a correlation between β-CAS activity and fungal resistance of the different lines examined (Geddes *et al.*, 2008; Zhang *et al.*, 2013). Moreover, exogenously applied HCN has been shown to enhance rice defense against the fungus *Magnaporthe grisea*, whose production is triggered by a hypersensitive reaction (HR) (Iwai *et al.*, 2006; Seo *et al.*, 2011). The underlying mechanism of cyanide production in plant immunity against these kinds of pathogens is very complex and requires further study.

In addition to its action in defense, cyanide also plays an important role during compatible and incompatible plant–bacteria interactions. Endogenous HCN content is oppositely regulated in virulent or avirulent interactions with the hemibiotrophic bacterium *P. syringae*. During an incompatible interaction between Arabidopsis plants and the avirulent *P. syringae* pv. tomato DC3000 avrRpm1 (Pst DC3000 avrRpm1), there is an early decrease in the expression of *CAS-C1* that leads to a transient increase in the concentration of HCN, which, analogous to the oxidative burst occurring at early stages of the infections, can be considered an HCN burst (Garcia *et al.*, 2013, 2014, 2019).

**Fig. 2.**
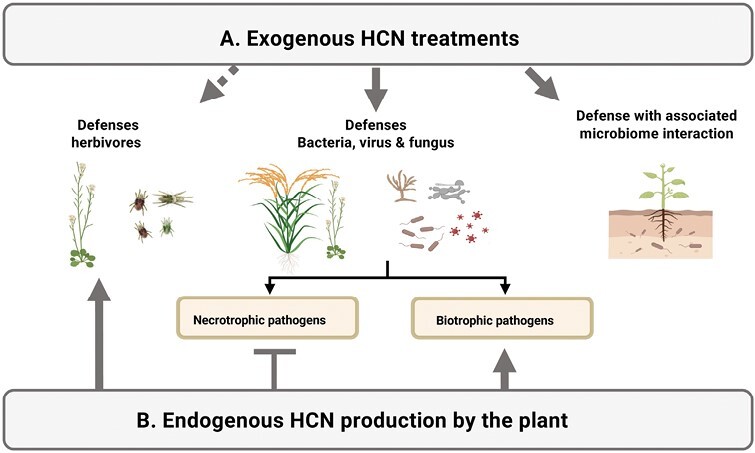
HCN and plant defense. (A) Exogenous treatments with HCN or the production of HCN by the plant-associated cyanogenic microbiome in the rhizosphere activate plant defense against a wide range of pathogens. (B) Plant endogenously produced HCN and HCN-producing molecules have been shown to promote plant defense against herbivores and biotrophic pathogens.

As mentioned above, a wide range of organisms, such as fungi, bacteria, lichens, millipedes, arthropods, and insects, are also cyanogenic (Zagrobelny *et al.*, 2008). In this context, HCN is also implicated in the plant-associated microbiome in the rhizosphere. Cyanogenic rhizobacteria produce HCN, which can also be used as an effective herbicide to inhibit the growth of weed seedlings in *Lactuca sativa* (Kremer and Souissi, 2001), an alternative to the toxic herbicides in current use. On the other hand, strong growth inhibition has been observed in various pathogenic fungi, weeds, insects, termites, and nematodes by the effect of cyanogenic bacteria (Sehrawat *et al.*, 2022). Numerous *Trichoderma* species show antagonism against phytopathogenic microorganisms producing HCN, siderophores, antibiotics, and fungal cell wall-lysing enzymes such as cellulases, ligninases, chitinases, and proteases (Jamil, 2021; Saeed *et al.*, 2021). HCN produced by *Trichoderma* acts as a defense regulator and inhibitor against different phytopathogens, including *Aspergillus niger*, *A. flavus*, *Fusarium oxysporum*, and *Alternaria alternata* (Blumer and Haas, 2000; Cucu *et al.*, 2019; Zain *et al.*, 2019; Jamil, 2021). In fact, other signaling molecules, such as NO, which has chemical characteristics very similar to HCN, have been suggested to be further involved in the induced systemic resistance of plants. Pescador *et al.* (2022) reported that NO of *Trichoderma* fungi causes induced systemic resistance in Arabidopsis plants and is effective against a wide range of pathogens. Therefore, the use of HCN produced by bacteria and fungi as a biocide may be a pathway for its use in sustainable agriculture (Rijavec and Lapanje, 2016; Sendi *et al.*, 2020; Sehrawat *et al.*, 2022). Figure 2 represents a scheme of the implication of HCN in plant defense.

## Mechanisms of action of HCN

The measured HCN content in plant tissues ranged from 25–150 ppb in Arabidopsis leaves and roots to 25–1000 ppm in cyanogenic plants such as cassava and bamboo (Haque and Bradbury, 2002). In cyanogenic plants, the release of cyanide depends on the action of hydrolytic enzymes (β-glucosidases) to break down the cyanoglycoside (Cressey and Reeve, 2019).

**Fig. 3.**
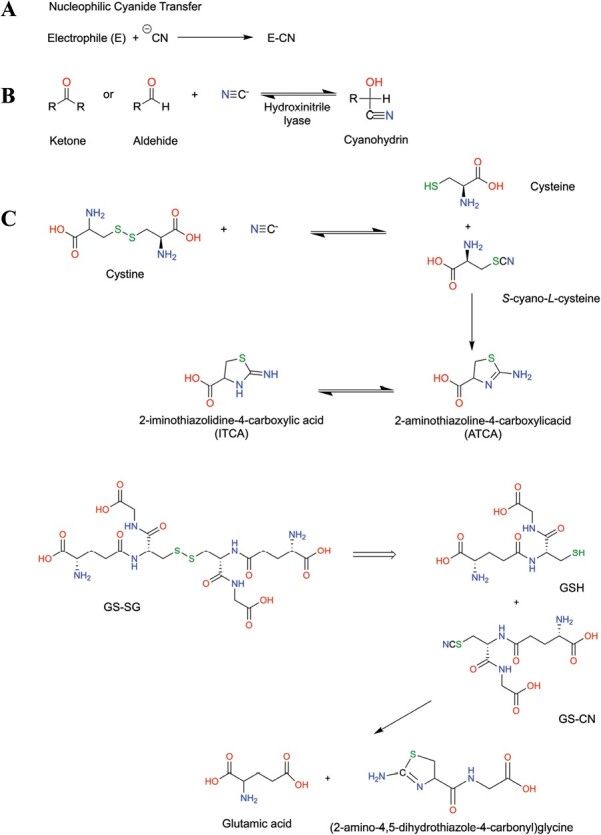
Cyanide reactivity with small molecules. (A) Schematic reaction of cyanide with electrophilic molecules. (B) Cyanohydrin formation by reaction with ketone and aldehyde molecules. (C) Cyanide reaction with disulfide bridges of cystine and oxidized glutathione.

The function of HCN as a signaling molecule, in a similar way to other signaling molecules such as H_2_S, must lie in the transient accumulation of cyanide and its chemical reactivity with other biomolecules. At least three mechanisms of action can be suggested to explain the signaling role of HCN in eukaryote cells (Fig. 3): (i) reaction with other small biomolecules; (ii) binding with metal centers and metalloproteins; and (iii) modification of protein cysteines to form the corresponding thiocyanate (Prot-S−C≡N).

HCN is weakly acidic, with a p*K*a of 9.2, and thus ionizes in aqueous solution to give the cyanide anion N≡C:^−^. The lone pair of electrons and the negative charge in the carbon atom make the cyanide anion a strong nucleophile that easily reacts with molecules by nucleophilic substitution reactions (Fig. 3A); thus, cyanide easily reacts with carbonyl compounds, especially aldehydes and ketones, to form cyanohydrins or hydroxynitriles (Fig. 3B). Cyanogenic plants contain the hydroxynitrile lyase enzyme that catalyzes the opposite reaction and breaks down cyanohydrin to release cyanide and aldehyde or ketone, and this reaction is a self-defense mechanism to protect plants containing cyanogenic glycosides from microbial and insect attack (Zagrobelny *et al.*, 2008). Although mainly described in cyanogenic plants, HNL activity has also been described in Arabidopsis to act in response to spider mite infestation (Arnaiz *et al.*, 2022). Cyanide can also react with cystine and mixed disulfides in general by nucleophilic attack on sulfur to yield a thiol and a thiocyanate, which further react to form more stable thiazoline heterocyclic compounds; thus, cystine reacts with cyanide to form cysteine and 2-aminothiazoline-4-carboxylic acid (ATCA) or its tautomer 2-iminothiazoline-4-carboxylic acid (ITCA) (Fig. 3C) (Boyde, 1968). These thiazolines can be detected in urine samples from humans with high dietary intake of cyanide and are used in forensic science (Lundquist *et al.*, 1995; Nishio *et al.*, 2022). Similarly, the oxidized form of glutathione (GSH), the most abundant thiol in eukaryote cells, can also react in the same way with cyanide and form reduced GSH and cyano-glutathione (GS-CN), which can cycle between the N-terminus of the cysteine residue and the CN bond, which cleaves the γ-glutamyl-cysteine peptide bond to produce the corresponding 2-aminothiazolidine (ATOEA) (or the tautomer 2-iminothiazolidine) and glutamic acid (Degani and Patchornik, 1974). The analysis of ATOEA in plasma has also been used as a biomarker of cyanide exposure (Gyamfi *et al.*, 2019). The presence of ATCA or ATOEA in plants has not been analyzed thus far, but the abundance of GSH in plant cells makes it plausible that it may act as a cyanide-detoxifying molecule or, vice versa, that cyanide accumulation during ET burst may act by reducing GSH levels under stress conditions. Cyanide can also react with reduced sulfur species such as polysulfides and, in the presence of an oxidizing agent, it can react with certain oxidized forms of sulfur such as sulfite and thiosulfate to produce thiocyanate (Bartlett and Davis, 1958).

The cyanide anion reacts with transition metals to form M-CN bonds and generate complexes with coordination numbers from two to eight (Hanusa, 2005). Cyanide complexes are commonly formed by transition metals with oxidation states (+3) and (+2), including iron, copper, mercury, zinc, nickel, cadmium, cobalt, other transition group metals, and, of course, gold and silver. The capacity to form very stable metal complexes is the basis of cyanide toxicity since it can bind to heme *a*_*3*_ of cytochrome *c* oxidase, inhibiting the utilization of molecular oxygen (Keilin *et al.*, 1939); thus, cyanide affects complex IV and inhibits mitochondrial electron transport and ATP generation (Keilin *et al.*, 1939). Although cyanide can inhibit cytochrome *c* oxidase activity, it has been recently reported that at a low concentration, cyanide stimulates mitochondrial electron transport and ATP generation associated with the removal of inhibitory glutathionylation post-translational modification of the 30 kDa and 57 kDa subunits (Randi *et al.*, 2021). Cyanide is also able to interact with other heme proteins, such as hemoglobin and myeloperoxidase (MPO), which catalyze the oxidation of chloride, bromide, and thiocyanate to produce strongly oxidizing molecules such as hypochlorous acid (HOCl), hypobromous acid (HOBr), or hypothiocyanous acid (HOSCN) during the neutrophil respiratory burst (Rossifanelli *et al.*, 1964; Winterbourn *et al.*, 2016). Interestingly, MPO is one of the mammalian enzymes described that produces cyanide from glycine (Zgliczynski and Stelmaszynska, 1979). Nitrite and sulfite reductases are key nitrogen and sulfur assimilatory enzymes found in bacteria, fungi, and plants; they contain an Fe–heme siroheme linked to the [4Fe-4S] cluster, and their interaction with cyanide positively shifts the redox potential of the siroheme, altering the redox capability of the enzymes (Liu *et al.*, 2014). Other metalloproteins are also susceptible to interaction with cyanide *in vitro*, such as Fe-superoxide reductase (FeSOD), Cu,Zn-superoxide dismutase (Cu,ZnSOD), and Mn-superoxide dismutase (Clarkson *et al.*, 1989; Iqbal and Whitney, 1991; Shearer *et al.*, 2003). Plant photosynthesis is also very sensitive to cyanide, which is a potent inhibitor of electron transfer to PSI since it can bind Cu-plastocyanin (Berg and Krogmann, 1975).

The high affinity of cyanide for cobalamin or vitamin B12, a coordination complex of cobalt ions in the center of a corrin heterocyclic ring, makes this molecule a more effective and common antidote in cyanide poisoning (Borron *et al.*, 2006). In plant tissue, treatment with hydroxocobalamin can reverse the inhibition of root hair development due to cyanide accumulation in mutant lines defective in the cyanide detoxification enzyme β-cyanoalanine synthase (Garcia *et al.*, 2010).

The recognized gaseous signaling molecules carbon monoxide (CO), NO, and H_2_S exert their cellular effects by interacting with cellular and molecular targets. The three of them can react with transition metals to give metal complexes, but, in addition to other mechanisms, NO and H_2_S carry out their function through the post-translational modification of cysteine thiol residues in protein targets, such as *S*-nitrosylation and persulfidation, respectively. HCN may react with protein disulfide by nucleophilic displacement and form *S*-cyanylated cysteine residues. This modification can be detected in human plasma proteins, such as immunoglobulin G and serum albumin, after cyanide poisoning or in individuals who smoke (Fasco *et al.*, 2007; Grigoryan *et al.*, 2016). The presence of *S*-cyanylation in a wide variety of proteins was described in Arabidopsis wild-type plants as well as in a mutant line defective in the β-cyanoalanine synthase enzyme, which accumulated higher levels of cyanide (Garcia *et al.*, 2010). Using two different technical approaches, a set of 163 proteins were shown to be susceptible to *S*-cyanylation. The first approach took advantage of the described characteristic of *in vitro S*-cyanylated proteins that can be cleaved in two parts under alkaline conditions, the amino acid backbone from the N-terminus of the cyanylated cysteine residue on one side and the cycled iminothiazolidine-derived peptide with the C-terminus of the protein on the other (Catsimpoolas and Wood, 1966; Fasco *et al.*, 2007). Comparative analysis of the protein profiles in wild-type and *cas-c1* lines revealed 88 cyanylated proteins involved in important metabolic pathways, such as glycolysis and the TCA and Calvin cycles. Enolase 1 and 2, part of the phosphopyruvate hydratase complex, showed a higher (>100-fold) cyanylation change in cas-c1 compared with the wild type, with Cys346 being the modified residue (Garcia *et al.*, 2019). Alternative approaches used LC-MS/MS to analyze protein extracts from enriched mitochondrial preparations from root tissues and identified an additional 75 *S*-cyanylated proteins in roots. To date, a total of 163 *S*-cyanylated proteins have been described in plants (Garcia *et al.*, 2019). Some of the more interesting findings were that most of the enzymes of the *S*-adenosylmethionine cycle, including Met synthase 1 and 2 (MS1 and MS2) and DNA methyltransferase 2 (DMT2), were modified. This may have important signaling and regulatory aspects related to protein and DNA methylation. Indeed, MS1 and DNA methylation are related to immune priming (González and Vera, 2019), which is a status that can deploy defense mechanisms more rapidly and robustly following a pathogen infection, with a minimal cost to the physiology of plants because the defensive response is initiated only if necessary. This may result in noteworthy implications that require further attention.

## Conclusion

**Fig. 4.**
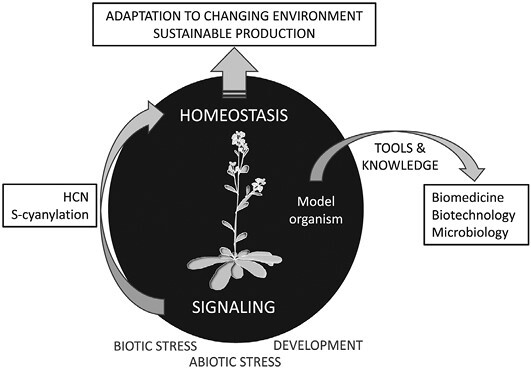
Conclusions and upcoming.

Information gained using model plants has changed the perception of HCN from a poison to a useful molecule that is able to generate a signal that transduces external (biotic or abiotic stress) or internal (development or other natural processes) signals in a response aiming to restore plant (or any organism) homeostasis and adapt to the new situation. Apart from agriculture (deepening the knowledge of the immune response and developmental processes in plants regulated by cyanide), generated tools and knowledge can be useful for exploring other areas, such as biomedicine (identifying new human pathophysiological processes regulated by HCN), microbiology (identifying pathophysiological processes in bacteria and fungi regulated by HCN), and biotechnology (manipulating biological processes by protein activity alteration by *S*-cyanylation) (Fig. 4).
